# Construction of polyomavirus-derived pseudotype virus-like particles displaying a functionally active neutralizing antibody against hepatitis B virus surface antigen

**DOI:** 10.1186/s12896-015-0203-3

**Published:** 2015-09-15

**Authors:** Milda Pleckaityte, Corinna M. Bremer, Alma Gedvilaite, Indre Kucinskaite-Kodze, Dieter Glebe, Aurelija Zvirbliene

**Affiliations:** Department of Immunology and Cell Biology, Institute of Biotechnology, Vilnius University, Graciuno 8, LT-02241 Vilnius, Lithuania; Institute of Medical Virology, National Reference Centre for Hepatitis B and D Viruses, German Centre for Infection Research, Justus-Liebig University of Giessen, Schubertstrasse 81, 35392 Giessen, Germany

## Abstract

**Background:**

Virus-like particles (VLPs) can be efficiently produced by heterologous expression of viral structural proteins in yeast. Due to their repetitive structure, VLPs are extensively used for protein engineering and generation of chimeric VLPs with inserted foreign epitopes. Hamster polyomavirus VP1 represents a promising epitope carrier. However, insertion of large sized protein sequences may interfere with its self-assembly competence. The co-expression of polyomavirus capsid protein VP1 with minor capsid protein VP2 or its fusion protein may result in pseudotype VLPs where an intact VP1 protein mediates VLP formation. In the current study, the capacity of VP1 protein to self-assemble to VLPs and interact with the modified VP2 protein has been exploited to generate pseudotype VLPs displaying large-sized antibody molecules.

**Results:**

Polyomavirus-derived pseudotype VLPs harbouring a surface-exposed functionally active neutralizing antibody specific to hepatitis B virus (HBV) surface antigen (HBsAg) have been generated. The pseudotype VLPs consisting of an intact hamster polyomavirus (HaPyV) major capsid protein VP1 and minor capsid protein VP2 fused with the anti-HBsAg molecule were efficiently produced in yeast *Saccharomyces cerevisiae* and purified by density-gradient centrifugation. Formation of VLPs was confirmed by electron microscopy. Two types of pseudotype VLPs were generated harbouring either the single-chain fragment variable (scFv) or Fc-engineered scFv on the VLP surface. The antigen-binding activity of the purified pseudotype VLPs was evaluated by ELISA and virus-neutralization assay on HBV-susceptible primary hepatocytes from *Tupaia belangeri*. Both types of the pseudotype VLPs were functionally active and showed a potent HBV-neutralizing activity comparable to that of the parental monoclonal antibody. The VP2-fused scFv molecules were incorporated into the VLPs with higher efficiency as compared to the VP2-fused Fc-scFv. However, the pseudotype VLPs with displayed VP2-fused Fc-scFv molecule showed higher antigen-binding activity and HBV-neutralizing capacity that might be explained by a better accessibility of the Fc-engineered scFv of the VLP surface.

**Conclusions:**

Polyomavirus-derived pseudotype VLPs harbouring multiple functionally active antibody molecules with virus-neutralizing capability may represent a novel platform for developing therapeutic tools with a potential application for post-exposure or therapeutic treatment of viral infections.

## Background

Viral structural proteins produced by heterologous expression may have the intrinsic ability to spontaneously self-assemble into highly organized particles – virus-like particles (VLPs) – that morphologically resemble viral capsid and represent a promising tool for protein engineering. The simplest VLPs are composed of a single viral structural protein, such as the assembly-competent envelope or capsid protein [1–4]. More complex recombinant VLPs can be generated by either genetic fusion or co-expression of the VLPs with an additional target protein or its segment. The co-expression of the assembly-competent viral structural protein with another viral structural protein or its fusion protein results in so-called pseudotype VLPs [3, 5]. Previous studies show that the major capsid protein VP1 of different polyomaviruses can be efficiently produced in yeast expression system in a form of VLPs [6] consisting of 72 pentamers of the VP1 protein [7]. Hamster polyomavirus (HaPyV) VP1 protein has been exploited for the generation of either chimeric VLPs harbouring foreign epitopes, or pseudotype VLPs when co-expressed with the minor capsid protein VP2 that binds within the central 5-fold cavity of each VP1 pentamer [8–11]. Moreover, it was demonstrated that HaPyV-derived pseudotype VLPs represent an efficient carrier for functionally active complex molecules, such as antibodies [12]. In these pseudotype VLPs, an intact VP1 protein is functioning as a carrier mediating VLP formation of both VP1 and the modified VP2 protein molecule. The C-terminal part of VP2 protein is necessary for its interaction with VP1 pentamer, therefore, the VP2 N-terminus can be shortened and used to join target molecules. The ratio of VP1:VP2 proteins in HaPyV capsid is 360:72 [7], consequently up to 72 chimeric VP2 molecules harbouring target molecules might be incorporated into one VLP. Based on the structure of polyomavirus capsids [13, 14], it was predicted that the inserted target sequence can be displayed on the surface of pseudotype VLPs. In contrast to chimeric VLPs that do not tolerate long inserts, this approach allowed efficient presentation of large-sized foreign protein sequences as demonstrated for the full-length human tumor-associated antigen Her2 [15, 16], prostate-specific antigen [17], green fluorescent protein [18], cellular marker p16^INK4A^ [19] and the antibody molecule [12]. The first example of a successful expression of a large recombinant antibody molecule on the surface of VLPs was generation of HaPyV-derived pseudotype VLPs harbouring the Fc-engineered single-chain antibody (scFv-Fc) specific to bacterial cytolysin vaginolysin (VLY). In this study, the scFv derived from hybridoma cell line producing high-affinity neutralizing antibody against VLY [20] was fused with human IgG1 Fc domain comprising the CH2 and CH3 domains and the hinge region [21]. To join the scFv-Fc molecule, the N-terminus of VP2 protein was deleted and replaced by the scFv-Fc molecule. It was demonstrated that the VLY-specific scFv-Fc fused with HaPyV VP2 protein and co-expressed with VP1 protein in the form of pseudotype VLPs was properly folded and exhibited a strong VLY-binding activity comparable to that of the parental monoclonal antibody (MAb). In contrast, the attempts to produce non-tagged, hexahistidine-tagged and alpha factor-tagged scFv-Fc proteins in yeast were unsuccessful due to their low yield, instability and a tendency to form insoluble aggregates [12, 22–24]. Therefore, an approach to display multiple functionally active antibody molecules on the surface of pseudotype VLPs that can be easily purified by density-gradient centrifugation may provide a promising alternative to other currently available methods for producing recombinant antibodies.

In the current study, we describe generation of polyomavirus-derived pseudotype VLPs harbouring surface-exposed scFv and scFv-Fc molecules specific to hepatitis B virus surface antigen (HBsAg). To our knowledge, this is the first example of pseudotype VLPs with an anti-viral activity.

## Results

### Generation in yeast of recombinant proteins harbouring anti-HBsAg molecule

**Fig. 1:**
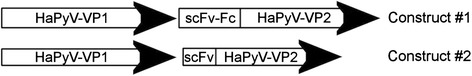
Schematic representation of recombinant proteins harbouring anti-HBsAg molecule

To generate pseudotype VLPs with surface-exposed HBsAg-specific antibody molecule, the gene encoding anti-HBsAg was fused with gene encoding modified HaPyV minor capsid protein VP2 and co-expressed together with the gene encoding HaPyV major capsid protein VP1. To join the anti-HBsAg molecule, the N-terminus of the VP2 protein was truncated by 100 amino acid (aa) residues. The production of both HaPyV VP1 protein and the modified VP2 protein was induced by adding galactose into yeast growth medium. Anti-HbsAg scFv comprising variable regions of mouse IgG in the orientation of VL-(G_4_S)_4_-VH was derived from the in-house produced hybridoma HB1 secreting neutralizing MAb against HBsAg (unpublished data). The cDNA sequences encoding VH and VL regions of anti-HBsAg MAb were deposited in GenBank under accession numbers KP972453 and KP972454. Two recombinant proteins harbouring HBsAg-specific antibody molecule were produced in yeast *S. cerevisiae* strain AH22-214p*:* one carrying the scFv-Fc molecule and one carrying the scFv without the Fc part (Table 1, Fig. 1).

**Table 1:** The list of recombinant plasmids for the expression of pseudotype VLPs in yeast *S. cerevisiae*

Plasmid	Protein	Short name
pFGG3-VP1/VP2-Fc-scFv	VP1/scFv-Fc-VP2	Construct #1
pFGG3-VP1/VP2-scFv	VP1/scFv-VP2	Construct #2

**Fig. 2:**
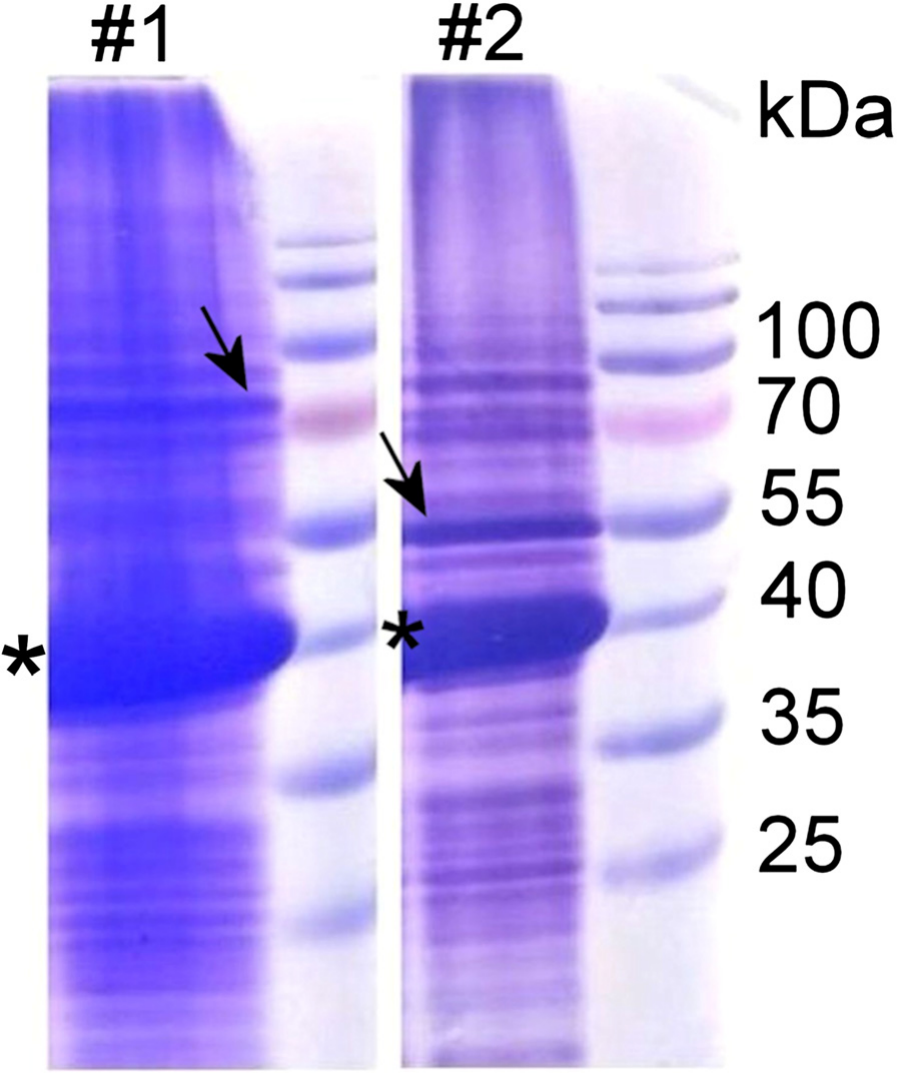
Analysis of recombinant proteins (constructs #1 and #2) partially purified on sucrose gradient by SDS-PAGE. The asterisk indicates the position of VP1 protein; the arrow indicates the position of VP2-fused protein. Prestained protein weight marker (Thermo Fisher Scientific) is presented on the right of each panel

After induction of the synthesis of recombinant proteins, the lysates of harvested yeast cells were examined by SDS-PAGE to evaluate the expression of VP1 and the scFv-Fc-VP2 fused proteins and then subjected to ultracentrifugation on sucrose density gradient. The analysis of partially purified recombinant proteins VP1/scFv-Fc-VP2 and VP1/scFv-VP2 (constructs #1, #2) by SDS-PAGE revealed a protein band of approximately 42 kDa that was identified as an intact VP1 protein (Fig. 2, the position indicated by an asterisk). Protein band of 78.4 kDa corresponding to the molecular mass of scFv-Fc-VP2 fusion protein was detected in the construct #1 (Fig. 2, the position indicated by an arrow). Protein band of 52.5 kDa corresponding to the molecular mass of scFv-VP2 fusion protein was detected in the construct #2 (Fig. 2, the position indicated by an arrow). As demonstrated by SDS-PAGE, the yield of recombinant proteins after the first purification step was similar and varied from 0.3 to 0.5 mg from 1 g of wet yeast biomass. The constructs #1 and #2 did not differ significantly in the expression levels of an intact VP1 protein.

### Purification and electron microscopy analysis of recombinant proteins harbouring anti-HBsAg molecule

**Fig. 3:**
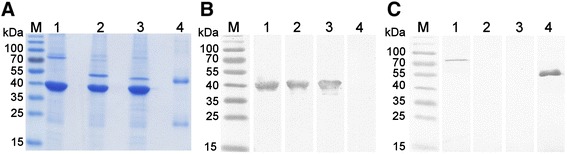
Analysis of recombinant constructs VP1/scFv-Fc-VP2 (construct #1, lane 1) and VP1/scFv-VP2 (construct #2, lane 2) purified on CsCl gradient by SDS-PAGE (**a**) and Western blot with the MAb 3D10 (**b**) and anti-human IgG antibody (**c**). Protein band corresponding to MW of 78.4 kDa represents scFv-VP2 fused protein (lane 1), protein band of 52.5 kDa represents scFv-VP2 fused protein (lane 2) and protein band of 42 kDa represents VP1 protein (lanes 1–3). The construct VP1/GFP-VP2 (lane 3) and purified human IgG (lane 4) are used as positive controls for Western blot

HaPyV-derived recombinant proteins VP1/scFv-Fc-VP2 (construct #1) and VP1/scFv-VP2 (construct #2) were successfully purified by the method adapted for the purification of VLPs using CsCl density centrifugation. The analysis of purified recombinant proteins by SDS-PAGE revealed the expected homogenous protein bands of 78.4 kDa and 42 kDa in case of construct #1 (Fig. 3a, lane 1) and homogenous protein bands of 52.5 kDa and 42 kDa in case of construct #2 (Fig. 3a, lane 2). Western blot with the HaPyV VP1-specific MAb 3D10 immunostained the 42 kDa protein band in both constructs #1 and #2 thus confirming the identity of the VP1 protein (Fig. 3b, lanes 1, 2). As a positive control for Western blot with VP1-specific MAb 3D10, the construct consisting of an intact HaPyV VP1 protein and the VP2 fuse with green fluorescent protein (eGFP) was used (VP1/GFP-VP2, Fig. 3b, lane 3). Western blot with HRP-labelled anti-human IgG antibody immunostained the 78.4 kDa band present in construct #1 thus confirming the identity of scFv-Fc-VP2 fusion protein (Fig. 3c, lane 1). No specific staining was obtained with construct #2 lacking the Fc part of human IgG (Fig. 3C, lane 2). As a positive control for Western blot with anti-human IgG, purified human IgG was used (Fig. 3C, lane 4).

As calculated from the SDS-PAGE with subsequent densitometry analysis, the VP1: scFv-Fc-VP2 ratio (w/w) in the VP1/scFv-Fc-VP2 (construct #1) is approximately 13:1, while the ratio the VP1: scFv-VP2 (w/w) in the VP1/scFv-VP2 (construct #2) is approximately 6:1. Based on the molecular weight (MW) of the VP2-fused proteins (78.4 kDa and 52.5 kDa, respectively), the calculated average number of the scFv-Fc-VP2 fused protein per one pseudotype VLP was 14 (construct #1), while the calculated number of the scFv-VP2 fused protein per one pseudotype VLP was 46 (construct #2).

**Fig. 4:**
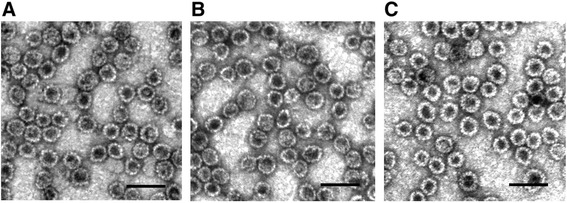
Electron microscopy pictures of CsCl-purified pseudotype VLPs stained with 2 % aqueous uranyl acetate solution and analysed by Morgagni 268 electron microscope. **a**, VP1/scFv-Fc-VP2 pseudotype VLPs (construct #1); **b**, VP1/scFv-VP2 pseudotype VLPs (construct #2), **c**, VP1/GFP-VP2 pseudotype VLPs (control). Scale bar – 100 nm

The purified recombinant proteins were negatively stained and subjected to electron microscopy (EM) analysis that confirmed an efficient self-assembly of both recombinant proteins to VLPs (Fig. 4). The pseudotype VLPs were approx. 45–50 nm in diameter, which corresponds to the size of intact HaPyV capsids and the unmodified recombinant HaPyV VP1-derived VLPs [8]. The size and shape of VP1/scFv-Fc- VP2 pseudotype VLPs (A) and VP1/scFv-VP2 pseudotype VLPs (B) were similar to that of VP1/GFP-VP2 pseudotype VLPs used as a positive control (C). The purified pseudotype VLPs were further analysed for the HBsAg-binding activity and the ability to neutralize HBV.

### HBV-neutralizing activity of pseudotype VLPs harbouring anti-HBsAg molecule

**Fig. 5:**
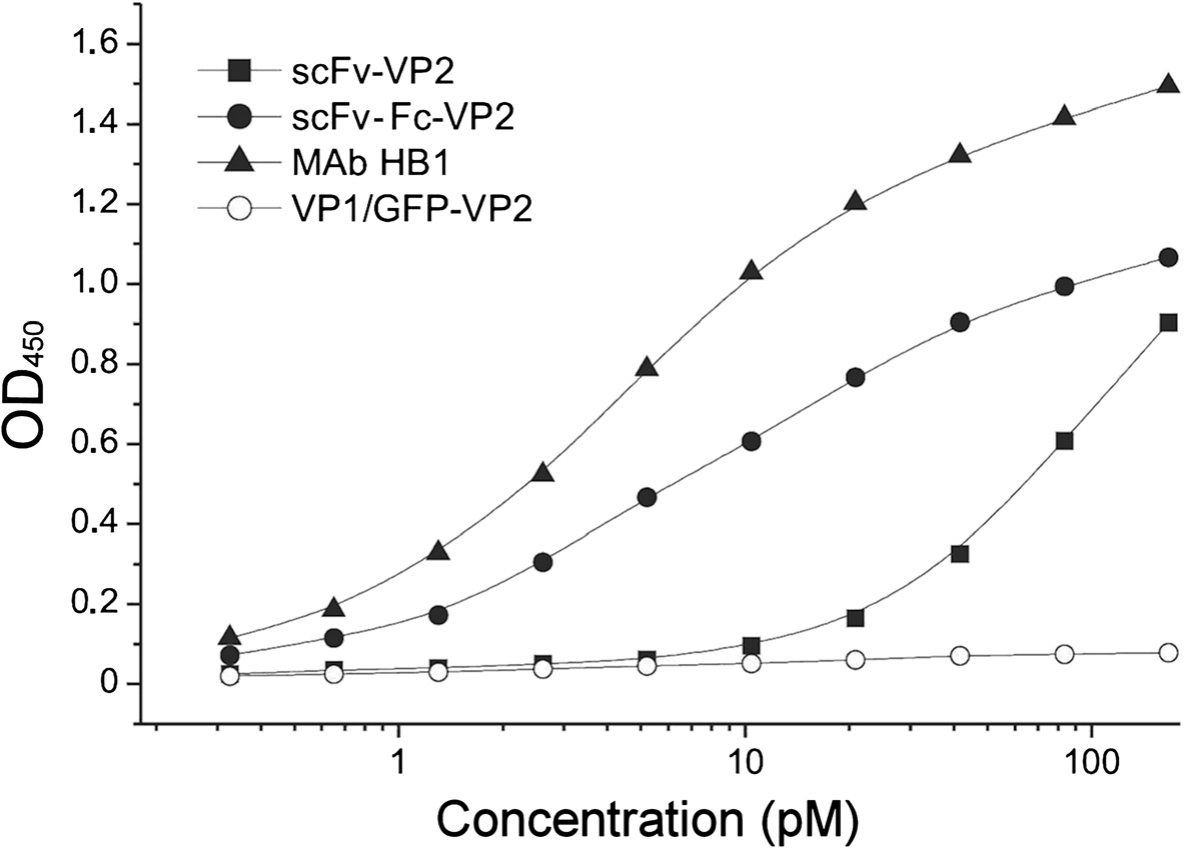
HBsAg-binding activity of pseudotype VLPs harbouring anti-HBsAg molecules determined by ELISA. Concentrations (pM) of anti-HBsAg fused proteins scFv-Fc-VP2 and scFv-VP2 displayed on the respective pseudotype VLPs are indicated. As a positive control, full-length parental MAb HB1 is used. As a negative control, VP1/GFP-VP2 pseudotype VLPs are used

**Fig. 6:**
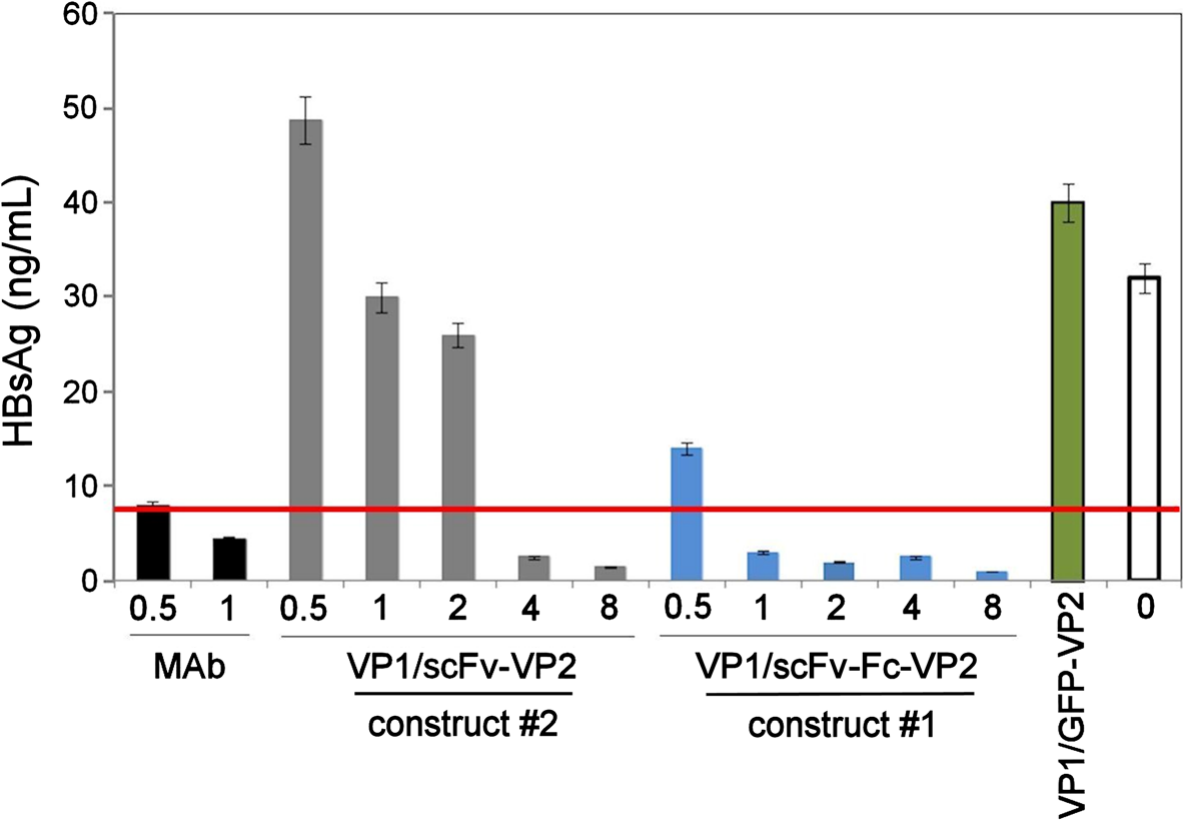
HBV-neutralizing activity of pseudotype VLPs harbouring anti-HBsAg molecule in comparison to the full length parental antibody (MAb). Concentrations (μg/mL) of anti-HBsAg fused proteins scFv-Fc-VP2 and scFv-VP2 displayed on the respective pseudotype VLPs (construct #1 and construct #2) are indicated. Highly purified HBV was preincubated with the indicated quantities of the respective proteins for 1 h at 16 °C and afterwards added to PTHs for 16 h at 37 °C. As a negative control, VP1/GFP-VP2 at concentration 8 μg/mL is used. Newly produced HBsAg of infected PTH cultures was determined on day 11. The red line indicates the Cut-off

Antigen-binding activity of the purified pseudotype VLPs was confirmed by an indirect ELISA using recombinant yeast-derived HBsAg (ayw) coated on the microtiter plate and the HaPyV VP1-specific MAb 3D10 as a detection antibody. Both anti-HBsAg fused proteins displayed on the respective pseudotype VLPs were reactive with the immobilized HBsAg (Fig. 5). The scFv-Fc-VP2 fused protein (construct #1) showed antigen-binding activity in a similar concentration range (starting from 1.5 × 10^−12^ M) as the parental MAb HB1 used as a positive control (starting from 0.7 × 10^−12^ M). Meanwhile, the scFv-VP2 fused protein (construct #2) was reactive with the plate-bound HBsAg when added at concentrations exceeding 24 × 10^−12^ M. The VP1/GFP-VP2 pseudotype VLPs used as a negative control did not show any reactivity with the immobilized antigen, which confirms the specificity of the ELISA (Fig. 5). Furthermore, the ability of the purified pseudotype VLPs to neutralize HBV was assayed *in vitro* using the HBV infection model of primary Tupaia hepatocytes (PTH) [25]. The neutralizing potency of the pseudotype VLPs was evaluated in comparison to the parental monoclonal antibody HB1 used as a positive control. The pseudotype VLPs were used at amounts that correspond to 0.5 μg/mL to 8 μg/mL of the anti-HBsAg fused proteins. The VP1/scFv-Fc-VP2 pseudotype VLPs containing the Fc part of human IgG1 (construct #1) showed higher HBV-neutralizing potency as compared to VP1/scFv-VP2 pseudotype VLPs (construct #2): 1 μg/mL (1.27 × 10^−10^ M) of scFv-Fc-VP2 fused protein displayed on the pseudotype VLPs (construct #1) were sufficient to induce complete HBV neutralization while 1 μg/mL (1.9 × 10^−10^ M) of the scFv-VP2 fused protein displayed on the pseudotype VLPs (construct #2) induced only partial HBV neutralization (Fig. 6). The observed HBV-neutralizing potency of the pseudotype VLPs VP1/scFv-Fc-VP2 (construct #1) containing 1 μg/mL (1.27 × 10^−10^ M) of scFv-Fc-VP2 fused protein was comparable to that obtained with 1 μg /mL (0.67 × 10^−10^ M) of the full-length parental MAb HB1. The specificity of the neutralization test was confirmed by the use of HaPyV-derived VP1/GFP-VP2 pseudotype VLPs (negative control) that did not induce HBV neutralization. The incomplete HBV neutralization by the construct #2 harbouring the scFv without the Fc part might indicate that the scFv molecule is less accessible on the surface of pseudotype VLPs as compared to the Fc-engineered scFv. The results of the HBV-neutralization test are consistent with the results of an indirect ELISA (Fig. 5) where the scFv-Fc-VP2 fused protein displayed on the VLPs showed higher antigen-binding activity as compared to the scFv-VP2 fused protein.

## Discussion

Recombinant VLPs are extensively used for various applications, from basic virus assembly and structure studies to the production of human and animal vaccines [3–5]. Due to their repetitive structure, VLPs represent an efficient carrier for B and T cell epitopes [4, 8, 10, 11]. Presentation of large protein sequences on VLPs may broaden the spectrum of potential VLP applications, however the long-sized inserts may interfere with the self-assembly competence of the viral carrier protein. To overcome this problem, pseudotype VLPs composed of different viral proteins can be generated. In the current study, the capacity of HaPyV-derived major capsid protein VP1 to self-assemble to VLPs and interact with the modified minor capsid protein VP2 has been exploited to generate pseudotype VLPs with surface-exposed multiple anti-HBsAg molecules. The pseudotype VLPs expressed in yeast and purified by density-gradient centrifugation were functionally active and showed a potent HBsAg-binding and HBV-neutralizing activity comparable to that of the parental MAb. The produced pseudotype VLPs harbouring surface-exposed anti-HBsAg molecules is the first example of anti-viral VLPs with virus-neutralizing capability. This approach may be further exploited to develop novel anti-viral tools for a post-exposure treatment of viral infections. Prevention of clinical disease in individuals already exposed to viral infection is an important issue of modern biomedicine. Recent advances in passive immunoprophylaxis of viral infections, such as rabies and human respiratory syncytial virus infection, are based on monoclonal antibody therapies [26, 27]. The *in vivo* studies have demonstrated the efficiency of neutralizing antibodies in established infections of influenza A, HIV, polioviruses, filoviruses and other viral infections [28–30]. The VLP approach combining the advantages of virus targeting by the surface-exposed antibody molecule and the capability of VLPs to encapsidate and deliver cargo, such as therapeutic nucleic acids or proteins [31, 32] may provide new possibilities for the potential development of effective anti-viral prophylactic and therapeutic strategies.

The current study demonstrates that antigen-binding capability of the pseudotype VLPs depends on the structure of the antibody molecule displayed on the VLPs. The anti-HBsAg molecule was presented on the VP2 protein in two different formats – either scFv alone or Fc-engineered scFv comprising CH2 and CH3 domains and the hinge region of human IgG1 [21]. The calculations based on the SDS-PAGE and densitometry data indicate that the VP2 protein fused with 473 aa-long scFv was incorporated into the pseudotype VLPs with higher efficiency (approx. 46 molecules per VLP in construct #2) as compared to 723 aa–long Fc-scFv (approx. 14 molecules per VLP in construct #1). However, construct #2 containing 3 times higher amount of anti-HBsAg scFv molecules showed much lower HBsAg-binding activity in ELISA and HBV-neutralizing potency in the *in vitro* HBV infection assay as compared to construct #1 harbouring Fc-engineered scFv molecules. The observed difference might be explained by a better accessibility of the Fc-engineered scFvs on the surface of VLPs. The quantity of anti-HBsAg molecules incorporated into the pseudotype VLPs of our study (14 anti-HBsAg Fc-scFv-VP2 molecules per one VLP) is consistent with a previous report where approx. 19 molecules of anti-VLY Fc-scFv-VP2 molecules were found to be displayed the pseudotype VLP [20]. Based on the resolved crystal structures of the virions of SV-40 and murine polyomavirus, a maximum of 72 VP2 molecules could be incorporated into one pseudotype VLPs as this corresponds to the number of VP1 protein pentamers that compose the VLP [7, 13, 14]. The current study clearly demonstrates that the number of large fusion proteins to be incorporated into a pseudotype VLP may vary according to the actual size of the modified VP2 molecule. Furthermore, the pseudotype VLPs harbouring multiple virus-neutralizing antibody molecules may represent a promising platform for therapeutic vaccines against acute or chronic viral infections. In case of HBV, construction of pseudotype VLPs combining different neutralizing antibodies directed against both HBsAg and preS1 sequences [33, 34] might provide a potent anti-viral tool with a broad HBV-neutralizing capacity. For human use, the scFvs derived from human MAbs would be of special interest [34] to avoid their enhanced immunogenicity after presentation on pseudotype VLPs.

## Conclusions

The current study demonstrates that HaPyV-derived pseudotype VLPs are suitable carriers for antibody molecules, both in the format of scFv and Fc-scFv. The pseudotype VLPs harbouring the Fc-scFv-VP2 molecule show higher antigen-binding activity and virus-neutralizing potency as compared to those harbouring the scFv-VP2 molecule. This difference might be explained by a more efficient exposure of the Fc-engineered scFv molecule on the surface of the pseudotype VLPs as compared to the scFv molecule.

The produced pseudotype VLPs harbouring surface-exposed anti-HBsAg molecules is the first example of anti-viral VLPs with virus-neutralizing capability.

The pseudotype VLPs harbouring multiple functionally active antibody molecules with virus-neutralizing capability represents a novel platform for the construction of therapeutic vaccines with a potential application for post-exposure or therapeutic treatment of viral infections. The VLP approach combining the advantages of virus targeting by the surface-exposed antibody molecule and the capability of VLPs to encapsidate and deliver cargo, such as therapeutic nucleic acids or proteins, might be further exploited for the development of novel efficient anti-viral strategies.

## Methods

### Construction of HBsAg-specific single-chain fragment variable (scFv)

All DNA manipulations were carried out according to standard procedures [35]. Enzymes and kits for DNA manipulations were purchased from Thermo Fisher Scientific (Vilnius, Lithuania). The anti-HBsAg scFv comprising variable regions of mouse IgG heavy (VH) [GenBank: KP972453] and light chains (VL) [GenBank: KP972454] in the orientation of VL-linker-VH was cloned and constructed as described previously [12]. Briefly, total mRNA was isolated from 3 × 10^6^ hybridoma cells (clone HB1) using GeneJET RNA Purification Kit (Thermo Fisher Scientific) according to manufacturer’s recommendations. Hybridoma clone HB1 was produced in-house using spleen cells of BALB/c mouse immunized with recombinant HBsAg (unpublished data). The first strand of cDNA was prepared using RevertAid™ H Minus First Strand cDNA Synthesis Kit (Thermo Fisher Scientific) according to manufacturer’s instructions. The cDNA corresponding to the variable regions of mouse IgG VH and VL was obtained by polymerase chain reaction (PCR) using sets of specific primers described previously [12, 36]. In the second PCR round, the VL and VH DNA fragments were fused to yield the construction VL-(G_4_S)_4_-VH.

### Construction of yeast expression plasmids

The anti-HBsAg scFv was displayed on the pseudotype VLPs either alone or fused with the Fc region of human immunoglobulin G1 encoded in pFUSE-hIgG1-Fc2 vector [21]. The resulting construction obtained by fusion of anti-HBsAg scFv to human Fc region was named scFv-Fc. Two yeast expression plasmids carrying either the scFv or scFv-Fc DNA fragment were generated (Table 1, constructs #1, #2). The recombinants were screened in *E. coli* DH10B cells.

The plasmid pFGG3-VP1/ VP2-Fc-scFv used for the co-expression of chimeric HaPyV-derived protein scFv-Fc-VP2 and the intact HaPyV VP1 protein (construct #1) were generated as described previously [12]. The construct encodes scFv-Fc molecule that is joined at the N–terminus of the VP2 protein truncated by 100 amino acid (aa) residues. The plasmid pFGG3-VP1/VP2-scFv encoding a similar construct without the Fc part (construct #2) was generated by cloning the scFv fragment into pFGG3-VP1/VP2Bg vector as described previously [12]. The plasmid pFGG3-VP1/VP2-eGFP was generated by cloning DNA fragment encoding green-fluorescent protein into the blunted XbaI and BglII sites of pFGG3-VP1/VP2Bg vector for fusion to modified HaPyV VP2-encoding gene.

### Yeast strains, growth media and cultivation conditions

The constructed plasmids pFGG3-VP1/VP2-Fc-scFv and pFGG3-VP1/VP2-scFv (Table 1), and pFGG3-VP1/VP2-eGFP were used for the transformation of *S. cerevisiae* strain AH22-214p (*a, leu2-3,112, his4-519, Δpep4*). Transformed yeast cells were cultivated as described previously [12]. Yeast biomass containing recombinant proteins was harvested by centrifugation and stored at −20 °C until purification. Yeast *S. cerevisiae* transformed with pFGG3 vector without any insert was used as a negative control.

### SDS-PAGE

Recombinant proteins were fractionated by electrophoresis on 12.5 % sodium dodecylsulfate-polyacrylamide gels (SDS-PAGE) and then either visualized by staining with Coomassie brilliant blue (Sigma–Aldrich, St. Louis, MO, USA). The quantities of loaded recombinant proteins were determined by densitometry using the ImageScanner III (GE Healthcare, Little Chalfont, UK) device supplied with the ImageQuantTL software. The determination of the quantities of VP1 and VP2-fused antibodies in the preparations of pseudotype VLPs was based on the comparison with the known quantities of VP1 and BSA proteins run in the same SDS-PAGE gel and scanned by the ImageScanner III. The molar quantities of VP1 and VP2-fused proteins in the samples of purified VP1/scFv-Fc-VP2 and VP1/scFv-VP2 constructs were calculated in accordance with their molecular weight (MW). The average number of VP2-fused proteins presented on one VLP was calculated as described previously [12]. Briefly, concentrations of purified pseudotype VLPs (mg/mL) were determined by Bradford assay [37] and the known quantities of each construct (1–3 μg per lane) were loaded on the SDS-PAGE gel. At least three scans of each VLP preparation were obtained. The determined quantities of VP1 protein (μg per lane) and VP2-fused proteins (μg per lane) were calculated in pmoles and then the number of protein molecules in both analyzed VLP preparations were determined based on the Avogadro constant. Assuming that 360 molecules of VP1 protein are needed for the assembly of one VLP [7] the calculated number of VP1 molecules was divided by 360 to determine the number of VLPs in the respective VLP preparation (VP1/scFv-VP2 VLPs and VP1/scFv-Fc-VP2 VLPs). The average number of VP2-fused molecules presented on one VLP was calculated by dividing the number of the respective VP2-fused molecules (scFv-VP2 and scFv-Fc-VP2) by the number of VLPs in the sample.

### Western blot

Recombinant proteins fractionated by SDS-PAGE were electrotransferred to Immobilon P membrane (Millipore, Bedford, MA, USA). The membranes were blocked with 5 % milk powder in phosphate-buffered saline (PBS) for 2 h at room temperature (RT) and rinsed in PBS with 1 % Tween-20 (PBST). For the detection of HaPyV VP1 protein, the membranes were incubated for 1 h at RT with monoclonal antibody (MAb) 3D10 [10]. The MAb was used at working dilution 1:1000 in PBST. The membranes were then incubated with horseradish peroxidase (HRP)-conjugated goat anti-mouse IgG (Bio-Rad) diluted 1:1000 in PBST. For the detection of scFv-Fc-VP2 fused protein, the membranes after blocking were incubated with HRP-conjugated rabbit anti-human IgG (Bio-Rad) diluted 1:2000 in PBST. After rinsing in PBST, the enzymatic reaction was developed using tetramethylbenzidine (TMB) chromogenic substrate (Sigma–Aldrich).

### Purification of pseudotype VLPs harbouring scFv-Fc

*S. cerevisiae* yeast biomass containing recombinant proteins was suspended and homogenized in DB450 buffer (450 mM NaCl, 1 mM CaCl2, 0.001 % Trition X-100, 0.25 M L-Arginine in 10 mM Tris/HCl-buffer pH 7.2) containing 2 mM PMSF, EDTA-free Complete Protease Inhibitor Cocktail (Thermo Fisher Scientific) and mechanically disrupted using French press. After centrifugation of yeast lysate, the supernatant containing recombinant proteins was collected and subjected to the ultracentrifugation on 30–65 % sucrose gradient and subsequently on CsCl density gradient ranging to buoyant densities from 1.23 to 1.38 g/mL as described previously [12]. The purified recombinant proteins were dialyzed against PBS and analysed by SDS-PAGE.

### Electron microscopy

The samples of purified recombinant proteins were placed on 400-mesh carbon coated palladium grids, negatively stained with 2 % aqueous uranyl acetate solution and examined by Morgagni 268 electron microscope (FEI Inc., Hillsboro, OR, USA).

### Indirect ELISA

Polystyrene microtiter plates (Nerbe plus, Winsen/Luhe, Germany) were coated with 100 μL per well of the recombinant HBsAg ayw (Abcam, Cambridge, UK) diluted in coating buffer (0.05 M sodium carbonate, pH 9.6) to a concentration of 5 μg/mL and incubated overnight at 4 °C. The coated plates were blocked with 2 % bovine serum albumin (BSA) for 1 h at RT and rinsed twice with PBST. Recombinant pseudotype VLPs were serially diluted in PBST, added to the wells and incubated for 1 h at RT. As a negative control, non-modified HaPyV VP1/VP2 VLPs were used. The plates were rinsed with PBST and incubated for 1 h with the MAb 3D10 diluted 1:1000 in PBST. The plates were rinsed with PBST and then incubated for 1 h with HRP-conjugated rabbit anti-human IgG (Bio-Rad) diluted 1:1000 in PBST. The plates were rinsed 5–7 times with PBST. Enzymatic reaction was visualized by the addition of 100 μL of ready-to-use TMB substrate (Sigma–Aldrich) to each well. After 10 min of incubation at RT, the reaction was stopped by adding 50 μL/well of 10 % sulphuric acid. The optical density (OD) was measured at 450 nm (reference filter 620 nm) in a microplate reader (Tecan, Groedig, Austria).

### HBV-neutralization assay on Tupaia hepatocytes

Neutralization test was performed as described previously [33]. Briefly, VLPs were preincubated with serum-derived purified HBV (ID326, genotype D) at a ratio of 10 HBV genomes/hepatocyte for 1 h at 16 °C in Tupaia hepatocyte medium (THM) and later incubated on primary *Tupaia belangeri* hepatocyte cultures (PTHs) for 16 h at 37 °C. As a positive control, the full-length MAb HB1 previously shown to exhibit the HBV-neutralizing activity in the *in vitro* assay was used. As a negative control, irrelevant pseudotype VLPs harbouring green-fluorescent protein (VP1/GFP-VP2) were used. The HBV-neutralization potency of the pseudotype VLPs was evaluated by measuring the concentration of the newly produced HBsAg in the culture supernatant on day 11th after infection. Animals were handled in a full accordance with ethical requirements. Permission to use primary hepatocytes from *Tupaia belangeri* was obtained from the Institutional Review Board of Justus-Liebig University of Giessen.
